# Accuracy of Bedside Methods for Preliminary Nasoenteric Tube Tip Localization in Intensive Care: A Prospective Diagnostic Study

**DOI:** 10.1111/nicc.70503

**Published:** 2026-04-21

**Authors:** Qianhui Yu, Yanmei Xie, Anshan Yu, Mingfang Xiao, Qingbang Zhong, Yueling He

**Affiliations:** ^1^ The First Clinical Medical College of Gannan Medical University Ganzhou China; ^2^ Department of Critical Care Medicine First Affiliated Hospital of Gannan Medical University Ganzhou China

**Keywords:** critical care, diagnostic tests, enteral nutrition, gastrointestinal intubation, point‐of‐care testing, routine

## Abstract

**Background:**

Postpyloric nasoenteric feeding reduces feeding intolerance and is recommended for critically ill patients. However, reliable confirmation of tip location remains challenging. Although radiography is the pre‐feeding gold standard, repeated use of radiography during insertion is impractical in resource‐limited intensive care units (ICUs).

**Aim:**

The aim of this study was to evaluate the accuracy of five bedside methods for preliminary nasoenteric tube tip localization against radiography. Secondary objectives included safety assessment and determining the optimal cut‐off for pH difference in aspirate.

**Study Design:**

This prospective, blinded diagnostic accuracy study was conducted in a tertiary ICU following the STARD 2015 guidelines, using a two‐stage assessment procedure: initial gastric verification followed by postpyloric advancement. All participants underwent five index tests performed by a trained senior nurse: pH difference in aspirate (comparing gastric and post‐advancement aspirate), pH testing of aspirate, auscultation of bubbling sounds, palpation for bubbling sounds and ultrasonographic gastric antrum assessment. Abdominal radiography served as the reference standard. The area under the curve (AUC) was calculated for each test with pairwise comparisons. Adverse events were monitored within 24 h. The optimal pH difference in aspirate cut‐off was determined using Youden's index.

**Results:**

Between October 2021 and January 2022, 60 patients were enrolled; postpyloric placement was confirmed in 47 (78.3%). Palpation for bubbling sounds (AUC 0.902), ultrasonographic gastric antrum assessment (AUC 0.863) and pH difference in aspirate (cut‐off ≥ 1, AUC 0.808) demonstrated superior accuracy compared with auscultation (AUC 0.622) and pH testing of aspirate (AUC 0.586) (all *p* < 0.05). The optimal pH difference in aspirate cut‐off was ≥ 1, yielding 100% sensitivity. No adverse events occurred.

**Conclusion:**

Palpation for bubbling sounds, ultrasonographic gastric antrum assessment and pH difference in aspirate are accurate bedside methods that can guide nurse‐led nasoenteric tube placement, reduce interim radiographs and reserve mandatory confirmation for pre‐feeding safety checks.

**Relevance to Clinical Practice:**

This study validates three accurate bedside methods—pH difference in aspirate, palpation for bubbling sounds and ultrasonographic gastric antrum assessment—that can be integrated into nurse‐led protocols to guide nasoenteric tube placement in resource‐limited ICUs, reducing reliance on interim radiographs while maintaining patient safety.

## Impact Statement

- What is known about this topic
  ○ Blind bedside placement of nasoenteric tubes is common in resource‐limited ICUs.
  ○ Accurate postpyloric tip localization is essential for successful enteral nutrition.
  ○ Conventional methods (auscultation and single pH) have significant limitations.
  ○ Radiography is the gold standard but impractical for repeated use during insertion.
- What this paper adds?
  ○ Validates three practical bedside methods: palpation for bubbling sounds, ultrasonographic gastric antrum assessment and pH difference in aspirate (threshold ≥ 1).
  ○ Palpation for bubbling sounds and ultrasonographic gastric antrum assessment achieved high diagnostic accuracy (AUC 0.902 and 0.863).
  ○ pH difference in aspirate (≥ 1) achieved 100% sensitivity, serving as an effective screening tool.
  ○ After structured training, nurses can reliably perform these techniques.
  ○ Combined use reduces interim radiographs while maintaining safety per ASPEN guidelines.

## 1 Introduction

Critically ill patients often develop feeding intolerance during gastric tube feeding, associated with increased pneumonia risk, mortality and prolonged ICU stays [1, 2, 3, 4]. Postpyloric feeding demonstrates superior outcomes and is recommended for high‐risk populations [5, 6]. Success depends on accurate nasoenteric tube (NET) tip placement beyond the pylorus. This study aimed to evaluate five bedside methods for NET tip localization to support nurse‐led practice in resource‐limited ICUs.

## 2 Background

Accurate bedside NET tip confirmation–a core nursing task–remains clinically challenging. Methods range from resource‐intensive techniques (e.g., endoscopy, electromagnetic guidance) to blind insertion, the latter being particularly relevant in resource‐limited settings [7]. According to the 2016 ASPEN Safe Practices for Enteral Nutrition Therapy, abdominal radiography remains the pre‐feeding gold standard for confirming blindly placed enteral access devices, whereas auscultation alone is explicitly discouraged due to unreliability [8]. To minimize repeat radiographs during blind insertion, nurses need reliable guidance methods, reserving X‐ray for definitive confirmation [8, 9]. Auscultation and pH testing have poor specificity and are susceptible to interference [10]. Ultrasonography is accurate but often limited by cost and operator requirements [11]. A recent meta‐analysis of 15 RCTs confirmed that ultrasound guidance significantly improves success rates, positioning accuracy and reduces complications compared with blind insertion [12]. Emerging evidence suggests focused training enables nurse‐performed ultrasound [13]. However, validated, low‐cost bedside methods for nurse‐led placement in resource‐limited settings remain lacking.

## 3 Aims and Objectives

This study aimed to evaluate the diagnostic accuracy—including sensitivity, specificity and area under the curve (AUC)—of five bedside methods (pH difference in aspirate [pH difference, comparing gastric and post‐advancement aspirate], pH testing of aspirate [pH testing], auscultation of bubbling sounds [auscultation], palpation for bubbling sounds [palpation] and ultrasonographic gastric antrum assessment [ultrasound]) for preliminary NET tip localization during blind insertion, using abdominal radiography as the reference standard. Secondary objectives included assessing the safety of these methods and determining the optimal cut‐off value for pH difference using Youden's index.

## 4 Design and Methods

This prospective, blinded diagnostic accuracy study was conducted in accordance with the Standards for Reporting Diagnostic Accuracy Studies (STARD) 2015 guidelines [14].

### 4.1 Setting and Sample

The study was conducted in a 20‐bed general ICU of a tertiary hospital, with a nurse‐to‐patient ratio of 2.5:1 and a comprehensive primary nursing model. A senior nurse, who held a critical care nursing certification and had 10 years of ICU experience with critical care specialization, performed patient assessment and screening for study eligibility, followed by all NET insertions and then the five index tests. Consecutive adults requiring NET placement for enteral nutrition were enrolled. Inclusion criteria included age ≥ 18 years. Exclusion criteria were refractory hemodynamic instability (mean arterial pressure < 60 mmHg despite vasopressor support), contraindications to enteral feeding or enteral nutrition administered within 4 h prior to the procedure.

### 4.2 Data Collection and Procedures

#### 4.2.1 Materials and Equipment

The equipment used included a Spiral‐tip weighted NET (CH10‐145; Flocare Bengmark, Nutricia, Netherlands), a mobile X‐ray system (CARESTREAM DRX‐Revolution), and an ultrasound system equipped with C5‐1S convex (1.0–5.0 MHz) and L12‐4S linear (4.0–12.0 MHz) probes. For pH measurements, both universal (ranges 1–14, graduation 1.0) and specialized (ranges 0.5–5.0, 5.5–9.0, 9.5–13.0, graduation 0.5) pH indicator strips were employed. All procedures followed manufacturer specifications.

#### 4.2.2 NET Placement Procedure

All NET insertions were performed by the same senior nurse who, as described above, completed patient assessment, screening and subsequently the five index tests. After ≥ 4 h of fasting, blind insertion was performed with patients supine, head‐of‐bed elevated 30° and mandible tucked against the sternum [7, 10]. A two‐stage assessment was conducted: Initial gastric verification followed by postpyloric advancement.

Stage 1—**Gastric verification:** Two nurses confirmed gastric placement by air bolus auscultation, aspirate pH ≤ 5.0 [15, 16, 17] and the absence of signs of aspiration. Following confirmation, metoclopramide (10 mg IV) and a 100 mL water bolus were administered.

Stage 2—**Postpyloric advancement and assessment:** The tube was then advanced to 75–85 cm. At this depth, all five index tests (pH difference, pH testing, auscultation, palpation and ultrasound) were performed sequentially. Definitive tube tip location was subsequently confirmed by abdominal radiography.

#### 4.2.3 Index Tests

The same senior nurse who inserted the tubes performed all five index tests. She had completed structured training in ultrasonography (4 h theory, 8 h supervised practice) and achieved competency (≥ 90% on written examination and successful landmark identification in 10 consecutive scans).

### 4.3 pH Difference

pH was measured at 45–55 cm (gastric) and 75–85 cm (post‐advancement). The difference was calculated. Cut‐off ≥ 1 determined post hoc via Youden's index (exploratory).

### 4.4 pH Testing

At 75–85 cm, postpyloric placement was indicated by pH ≥ 6.5 (pre‐specified) [18]. Inability to aspirate after 100 mL water bolus was predefined as indeterminate (excluded from pH‐based analyses but retained for others).

### 4.5 Auscultation

**FIGURE 1:**
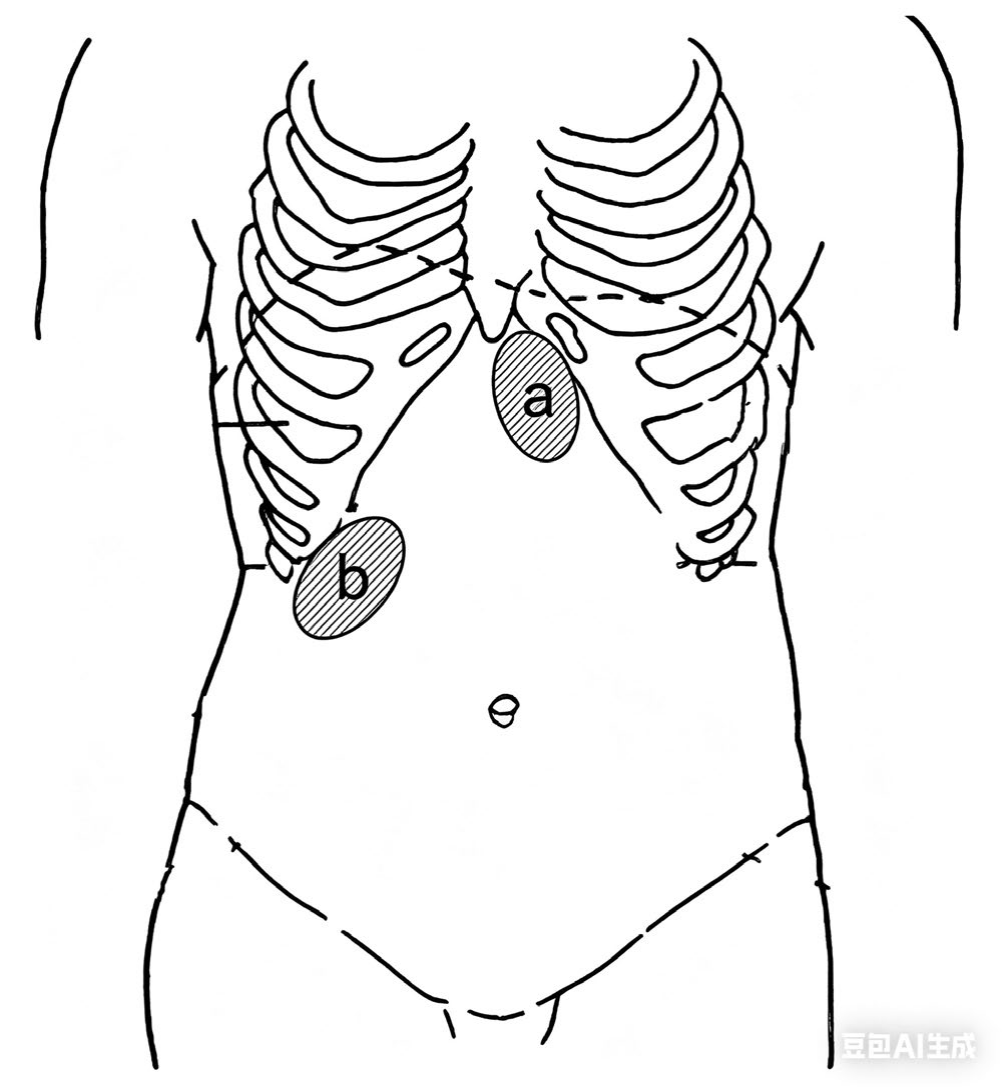
Auscultation/palpation site for bubbling sounds. (A) Site for auscultation/palpation of the gastric bubble and (B) over the duodenal bulb.

At 75–85 cm, an assistant administered a 10‐mL air bolus; the operator auscultated the gastric bubble (Figure 1A). A second bolus was then administered with auscultation over the duodenal bulb (Figure 1B). Louder, sharper duodenal sound indicated postpyloric placement.

### 4.6 Palpation

The same two‐step procedure was followed: After the initial 10‐ml air bolus with gastric palpation (Figure 1A), a second bolus was administered during palpation over the duodenal bulb (Figure 1B). Stronger vibration at the duodenal site indicated postpyloric placement. Absence of clear sound/vibration difference after two boluses was predefined as indeterminate.

### 4.7 Ultrasound

**FIGURE 2:**
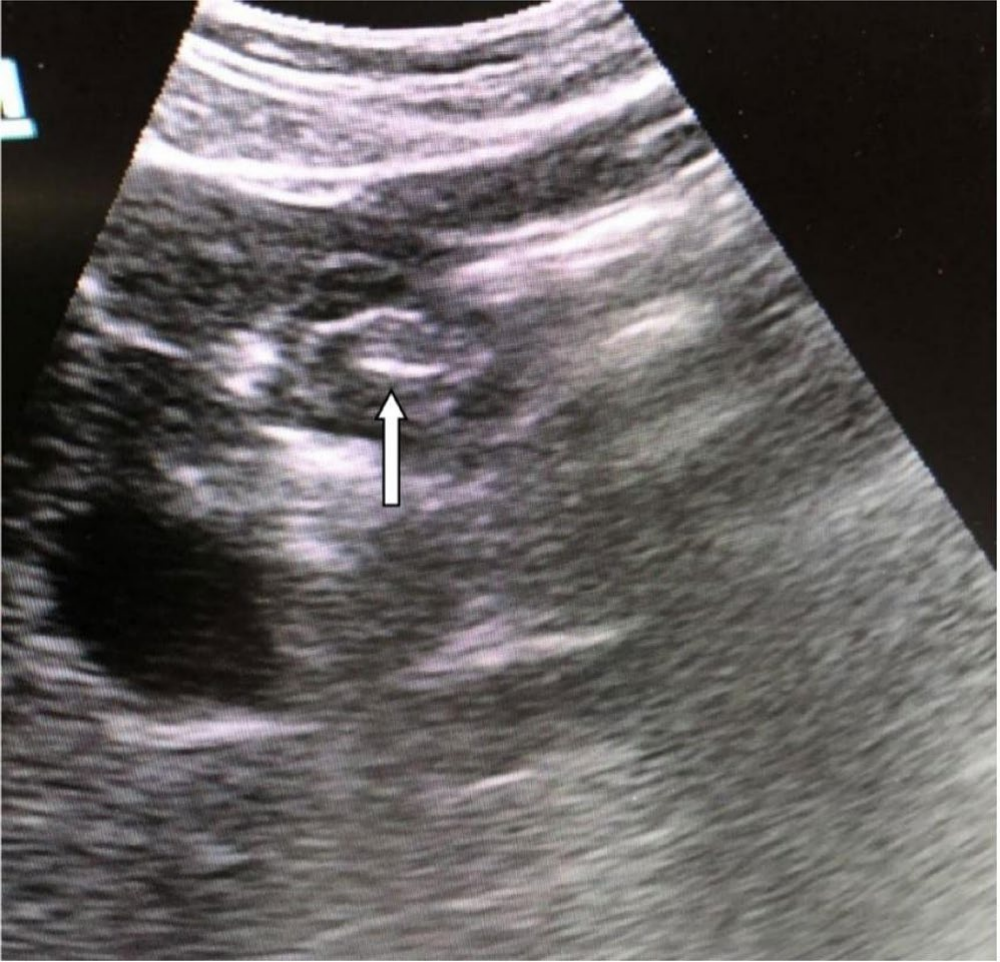
Arrow points to the characteristic ‘bright spot sign’ of the NET in the gastric antrum. NET, nasoenteric tube.

At 75–85 cm, the gastric antrum was visualized in the subxiphoid paramedian sagittal plane, sweeping to the right upper quadrant for short‐axis views, followed by a 90‐degree rotation of the probe for long‐axis visualization. Postpyloric placement was confirmed by the pathognomonic ‘bright spot sign’ in the gastric antrum [19] (Figure 2). Inability to identify the antrum or sign was predefined as an indeterminate result.

#### 4.7.1 Reference Standard

Abdominal radiography, the established reference standard for definitive confirmation of NET tip location [8, 9, 10], was performed immediately after ultrasound completion using a bedside mobile X‐ray system. Two board‐certified radiologists (each with ≥ 5 years of experience) independently verified tube position via the institutional Picture Archiving and Communication System (PACS). Postpyloric placement was confirmed by radiographic visualization of the tube tip distal to the pylorus. In cases where initial radiographic findings were indeterminate, supplementary cross‐sectional imaging (computed tomography/magnetic resonance imaging [CT/MRI]) was obtained. Persistent non‐diagnostic findings despite supplementary imaging were predefined as an indeterminate result.

#### 4.7.2 Adverse Events

Within 24 h post‐intubation, patients were monitored for aspiration, tube malposition and gastrointestinal perforation.

#### 4.7.3 Data Collection and Blinding

Data were collected using a standardized case report form (CRF) that captured demographics, clinical characteristics, index test results, reference standard outcomes and adverse events. No interventions occurred between the index tests and the reference standard; abdominal radiography was performed immediately after ultrasound completion using a bedside mobile X‐ray system, and the results were documented in the CRF upon interpretation by the radiologists. All assessments were pre‐specified, unless otherwise noted. The nurse performing the index tests was unaware of the radiographic results, and the radiologists interpreting the reference standard were blinded to the index test results.

### 4.8 Data Analysis

#### 4.8.1 Sample Size

Power analysis in PASS 11.0 (NCSS LLC) used the ‘Tests for Two ROC Curves’ procedure, with sample size calculations based on methods described by Jung [20] and Obuchowski and McClish [21]. Pilot data (*n* = 30) showed an AUC of 0.899 for palpation and 0.686 for auscultation. With a two‐sided α = 0.05, 90% power and an assumed 70% postpyloric success rate, 55 participants were needed. Accounting for 10% attrition, the target was *n* = 60.

#### 4.8.2 Handling of Missing and Indeterminate Data

Reference standard: Missing/indeterminate results would lead to exclusion from all analyses.

Index tests: Per‐test analysis–missing/indeterminate results excluded only that test's data for that participant.

#### 4.8.3 Statistical Analysis

Statistical analyses were performed using MedCalc 23.1.7 and PASS 11.0. Pre‐specified analyses included sensitivity, specificity, accuracy, positive predictive value (PPV), negative predictive value (NPV) with 95% CIs and AUC calculation with pairwise comparisons. The cut‐off for pH difference (≥ 1) was determined post hoc via Youden's index as an exploratory analysis. Continuous data: mean (SD); categorical: *n* (%). Two‐tailed *p* < 0.05 statistically significant.

### 4.9 Ethical Considerations and Trial Registration

The study protocol was approved by the Institutional Review Board on 14 October 2021. Written informed consent was obtained from all participants or legal representatives. The study was conducted in accordance with the Declaration of Helsinki and was reported following the STARD 2015 guidelines. The trial was registered with the Country Clinical Trial Registry (ChiCTR2100052117), and the full protocol is available at: https://www.chictr.org.cn/showprojEN.html?proj=135376.

## 5 Results

### 5.1 Participant Characteristics

**FIGURE 3:**
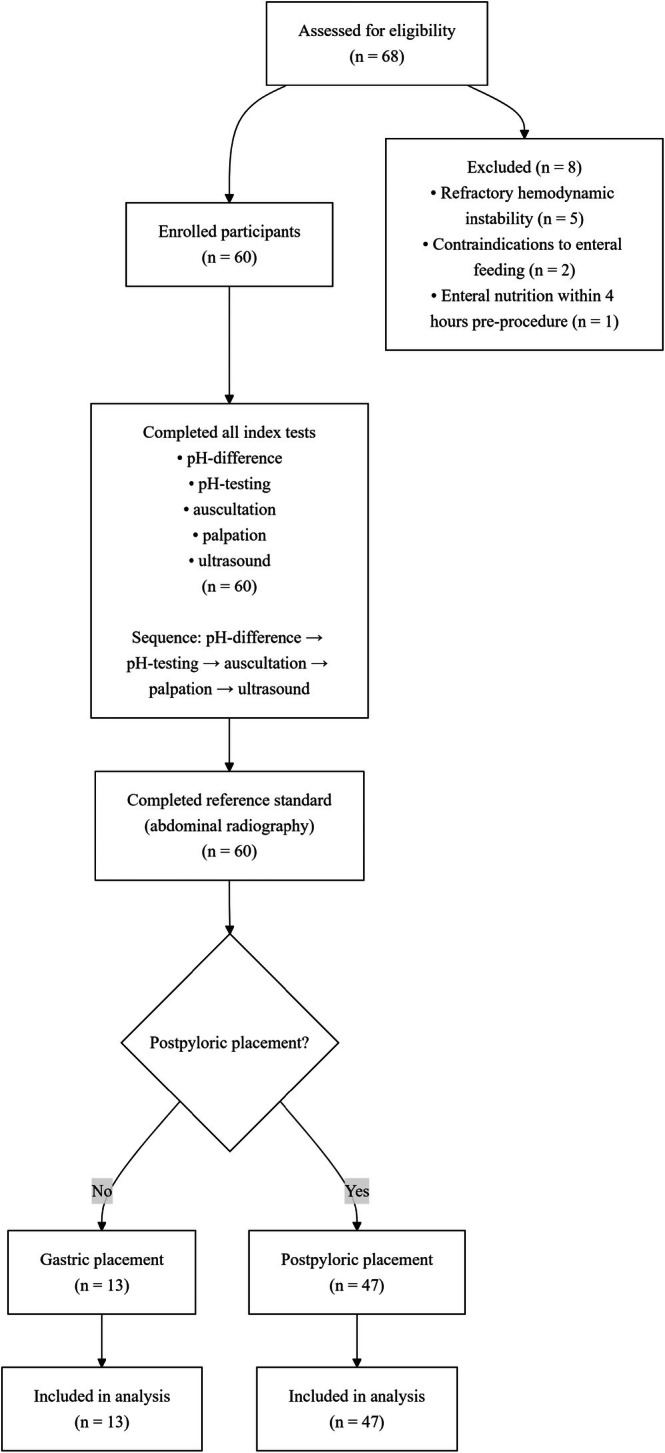
Participant flow diagram. Auscultation: auscultation of bubbling sounds; palpation: palpation for bubbling sounds; pH difference: pH difference in aspirate; pH testing: pH testing of aspirate; ultrasound: ultrasonographic gastric antrum assessment.

Between October 2021 and January 2022, all 60 enrolled participants completed assessments with no missing or indeterminate results. Among the 60 enrolled patients, the majority were male (81.7%), with a mean age of 58.9 years. Most participants (86.7%) were mechanically ventilated. Table 1 summarizes participant characteristics stratified by placement outcome. Radiographic confirmation demonstrated successful postpyloric placement in 47 patients (78.3%) and gastric placement in the remaining 13 (21.7%). Figure 3 illustrates the participant flow diagram.

**TABLE 1:** Participant characteristics by placement outcome (*N* = 60).

Characteristics	Postpyloric placement (*n* = 47)	Gastric placement (*n* = 13)	*χ* ^*2*^ */t*	*P*
Male, *n* (%)	38 (80.9%)	11 (84.6%)	0.097	0.755
Mechanical ventilation, *n* (%)	41 (87.2%)	11 (84.6%)	0.062	0.804
Age, years, mean (SD)	59.2 (15.8)	58.1 (17.9)	0.222	0.825
APACHE II, mean (SD)	22.8 (5.1)	24.1 (6.7)	−0.825	0.413
BMI, kg/m^2^, mean (SD)	22.4 (5.9)	23.9 (7.1)	−0.884	0.381
Placement difficulty factors, *n* (%)				
Gastric residual > 500 mL	3 (6.4%)	2 (15.4%)	1.199	0.273
BMI ≥ 28 kg/m^2^	3 (6.4%)	1 (7.7%)	0.030	0.862

### 5.2 Optimal Cut‐off Value for pH Difference

**FIGURE 4:**
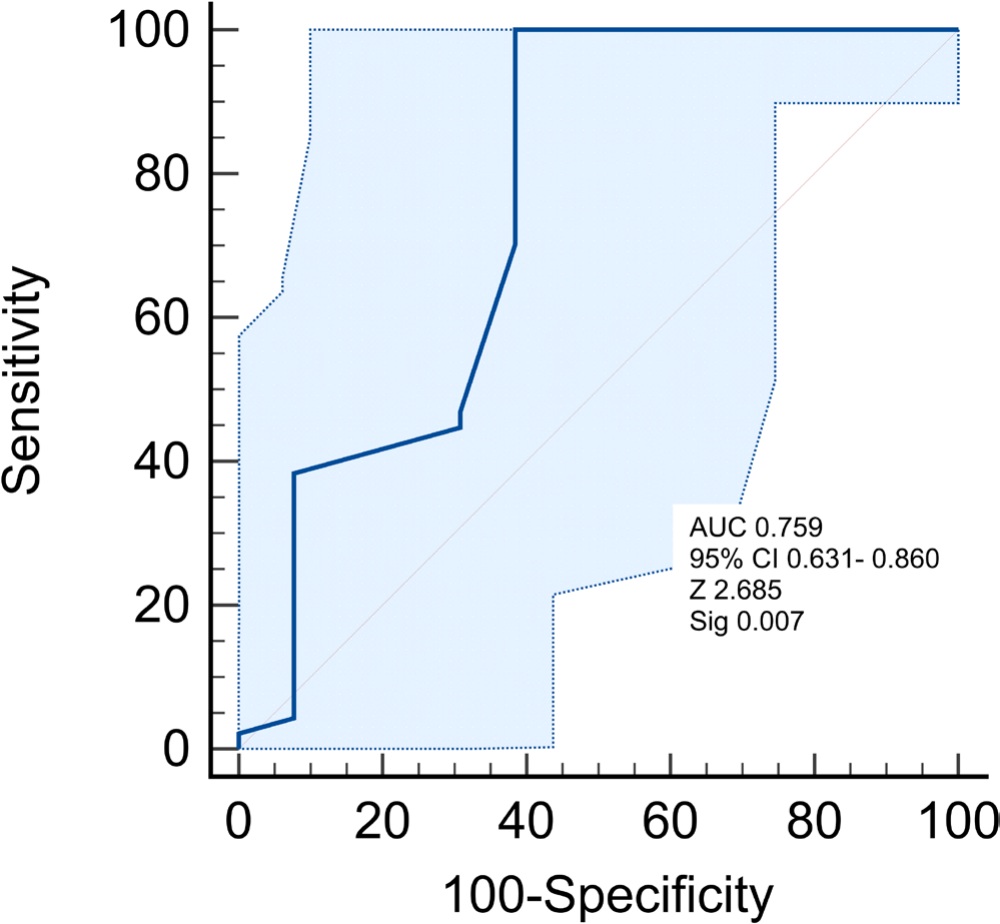
pH difference ROC curve. pH difference: pH difference in aspirate; AUC, area under the curve; ROC, receiver operating characteristic curve.

The AUC for pH difference was 0.759 (95% CI: 0.631–0.860) (Figure 4). Youden's index identified optimal cut‐off ≥ 1, yielding 100% sensitivity (95% CI: 92.2–100) and 61.5% specificity (95% CI: 32.3%–86.5%). At this cut‐off, the diagnostic performance expressed as (sensitivity + specificity)/2 was 0.808, which can be interpreted as the ‘cut‐off‐specific AUC’. The latter value is used in the subsequent analyses and summary tables.

### 5.3 Diagnostic Performance of Index Tests

**FIGURE 5:**
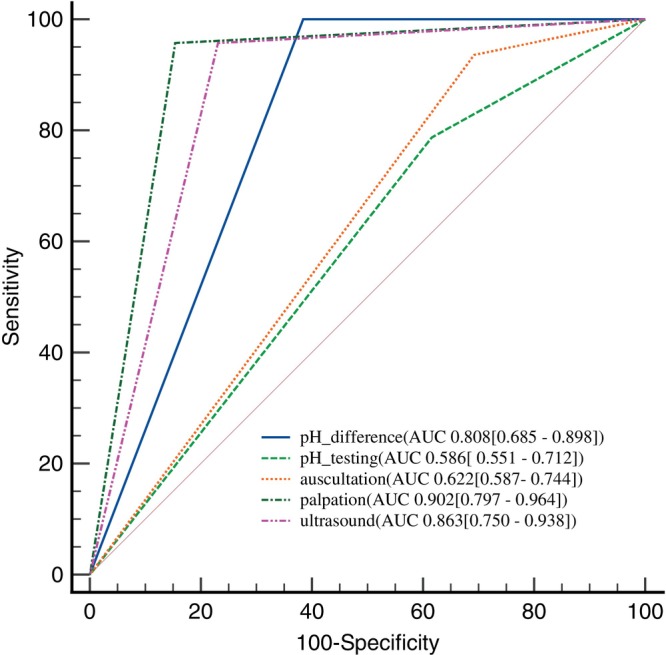
ROC curves of index tests. Auscultation: auscultation of bubbling sounds; palpation: palpation for bubbling sounds; pH difference: pH difference in aspirate; pH testing: pH testing of aspirate; ultrasound: ultrasonographic gastric antrum assessment; AUC: area under the curve; ROC: receiver operating characteristic curve.

Table 2 and Figure 5 summarize performance. Pairwise AUC comparisons (Table 3) showed that palpation (AUC 0.902), ultrasound (AUC 0.863) and pH difference (cut‐off ≥ 1, AUC 0.808) significantly outperformed auscultation (AUC 0.622) and pH testing (AUC 0.586) (all *p* < 0.05).

**TABLE 2:** Diagnostic performance of all index tests (*N* = 60).

Index tests	Sensitivity (%, 95% CI)	Specificity (%, 95% CI)	Accuracy (%, 95% CI)	AUC (95% CI)	PPV (%, 95% CI)	NPV (%, 95% CI)
pH difference	100 (92.2–100)	61.5 (32.3–86.5)	91.7 (81.4–97.2)	0.808 (0.685–0.898)^a^	90.4 (79.4–97.1)	100 (63.5–100)
pH testing	78.7 (65.3–89.4)	38.5 (14.3–68.4)	70.0 (57.3–81.1)	0.586 (0.551–0.712)	82.2 (68.4–92.2)	33.3 (12.4–62.3)
Auscultation	93.6 (83.4–99.2)	30.8 (9.5–61.3)	80.0 (68.1–89.4)	0.622 (0.587–0.744)	83.0 (70.4–92.1)	57.1 (18.5–90.4)
Palpation	95.7 (84.6–99.5)	84.6 (54.6–98.1)	93.3 (84.3–98.1)	0.902 (0.797–0.964)	95.7 (85.4–99.3)	84.6 (55.6–98.1)
Ultrasound	95.7 (85.4–99.3)	76.9 (46.2–95.0)	91.7 (81.4–97.2)	0.863 (0.750–0.938)	93.8 (82.2–99.3)	83.3 (52.4–98.1)
Palpation and ultrasound	93.6 (83.5–99.2)	92.3 (64.3–100)	93.3 (84.3–98.4)	0.930 (0.833–0.980)	97.8 (88.3–100)	80.0 (52.3–96.4)

Abbreviations: AUC, area under the curve; auscultation, auscultation of bubbling sounds; NPV, negative predictive value; palpation, palpation for bubbling sounds; pH difference, pH difference in aspirate; pH testing, pH testing of aspirate; PPV, positive predictive value; ultrasound, ultrasonographic gastric antrum assessment.

^a^ Based on cut‐off ≥ 1.

**TABLE 3:** Pairwise comparison of AUC among index tests (*N* = 60).

Index tests	ΔAUC (95% CI)	*z*	*p*
Palpation vs. auscultation	0.280 (0.134–0.426)	3.766	< 0.001
Palpation vs. pH testing	0.316 (0.159–0.473)	3.946	< 0.001
Ultrasound vs. auscultation	0.241 (0.096–0.387)	3.249	0.001
Ultrasound vs. pH testing	0.277 (0.124–0.431)	3.534	< 0.001
pH difference vs. pH testing	0.222 (0.049–0.395)	2.509	0.012
pH difference vs. auscultation	0.186 (0.011–0.361)	2.081	0.037
Palpation vs. ultrasound	0.038 (−0.100–0.176)	0.549	0.583
Palpation vs. pH difference	0.094 (−0.071–0.260)	1.115	0.265
Ultrasound vs. pH difference	0.056 (−0.050–0.162)	1.027	0.304
Auscultation vs. pH testing	0.036 (−0.153–0.225)	0.374	0.708

Abbreviations: AUC, area under the curve; auscultation, auscultation of bubbling sounds; palpation, palpation for bubbling sounds; pH difference, pH difference in aspirate; pH testing, pH testing of aspirate; ultrasound, ultrasonographic gastric antrum assessment.

**FIGURE 6:**
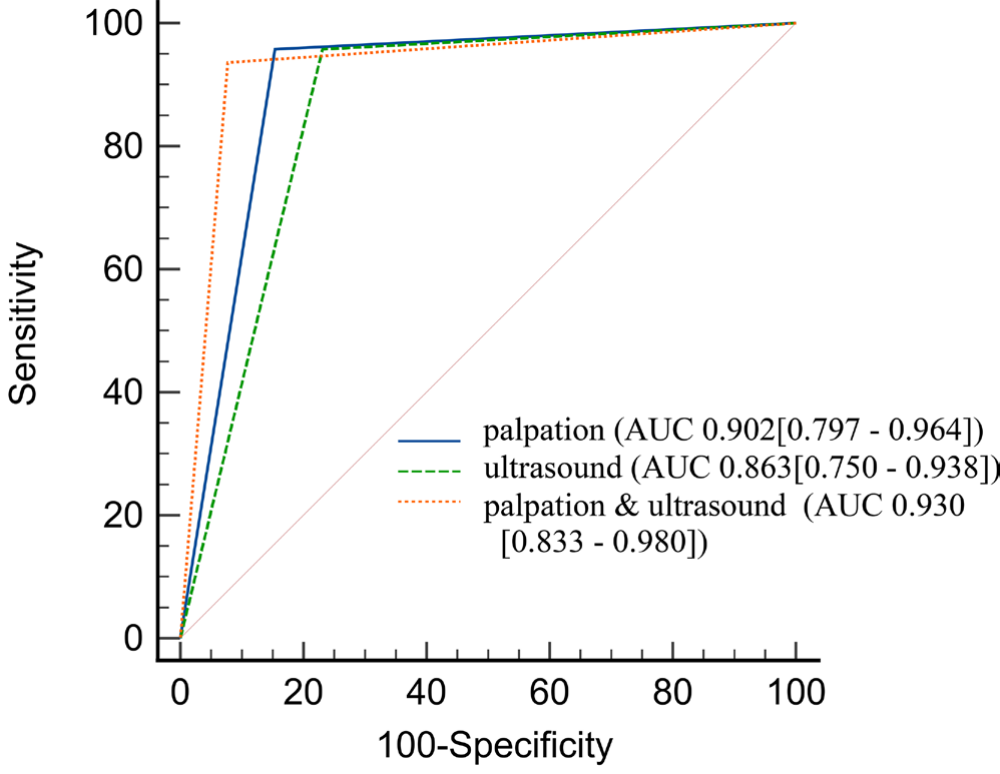
ROC curves for index tests palpation, ultrasound and their exploratory combination. Palpation: palpation of bubbling sounds; ultrasound: ultrasonographic gastric antrum assessment; AUC, area under the curve; ROC, receiver operating characteristic curve.

An exploratory serial combination of palpation and ultrasound (both positive) achieved the highest specificity (92.3%) and an AUC of 0.930 (Figure 6). AUC difference from individual tests was not statistically significant.

### 5.4 Adverse Events

No immediate or delayed adverse events occurred within 24 h post‐procedure.

## 6 Discussion

This prospective diagnostic accuracy study evaluated five bedside methods for preliminary NET tip localization against abdominal radiography as the reference standard. Palpation for bubbling sounds (AUC 0.902), ultrasonographic gastric antrum assessment (AUC 0.863) and pH difference in aspirate (cut‐off ≥ 1, AUC 0.808) demonstrated superior accuracy compared with auscultation (AUC 0.622) and single pH testing (AUC 0.586). The optimal pH difference cut‐off was ≥ 1, yielding 100% sensitivity.

The postpyloric success rate in this study was 78.3%. Contrary to previous reports [22, 23], factors associated with placement difficulty—namely high gastric residual volume and elevated BMI—did not significantly affect success in this cohort. The lack of association with gastric residual volume likely reflects active advancement following metoclopramide administration rather than passive migration [24]. Regarding BMI, our use of blind insertion with diagnostic (not guidance) ultrasound explains the absence of a BMI effect, in contrast to ultrasound‐guided placement where visualization may be impaired [25]. These results suggest that with an active, protocol‐driven insertion strategy, traditional risk factors may be less consequential, supporting the broader applicability of nurse‐led protocols.

pH testing showed poor utility (AUC 0.586), confirming the limitations of a single measurement due to buffering by feeds or medications. In contrast, pH difference proved robust (cut‐off ≥ 1, AUC 0.808), with ≥ 1 threshold achieving perfect sensitivity (100%) and NPV, making it an excellent screening tool to rule out gastric placement. The high NPV supports confident tube advancement when a pH increase is observed. However, modest specificity (61.5%) means that a pH difference < 1 does not reliably exclude postpyloric placement, necessitating confirmation by other methods. Our findings align with prior literature demonstrating pH difference is less influenced by enteral feeding or acid suppression than single pH measurement [18, 26, 27]. This simple, low‐cost, objective rule‐out criterion is well‐suited for nurse‐led bedside decision‐making.

Palpation demonstrated superior diagnostic capability (AUC 0.902, sensitivity 95.7%, specificity 84.6%). Although direct comparisons are limited by scarce palpation research, its performance can be contextualized against auscultation targeting the same site. A 2015 systematic review highlighted auscultation's poor specificity, consistent with our findings (AUC 0.622) [11]. Palpation's higher accuracy (ΔAUC 0.280, *p* < 0.001) likely reflects tactile vibration being less susceptible to acoustic dispersion and ambient noise. This suggests tactile feedback offers a more reliable physical signal for bedside confirmation.

Ultrasonographic gastric antrum assessment demonstrated strong diagnostic value (AUC 0.863, sensitivity 95.7%, specificity 76.9%), consistent with systematic review evidence [11] and original studies [19, 28]. Our results exceed those of Rigobello et al. [28] (sensitivity 79.0%, specificity 66.7% for nurse‐performed ultrasound), likely due to two factors: focused 12‐h training and anatomical simplification to a single landmark (the gastric antrum with the ‘bright spot sign’), reducing technical complexity. This finding aligns with Brotfain et al. [13], who showed ICU nurses with 4 h of training successfully verified tube placement, and Re et al. [29], confirming the gastric antrum as a key landmark. As the American Society for Parenteral and Enteral Nutrition (ASPEN) guidelines emphasize, ultrasound serves as a valuable adjunct during insertion, minimizing repeat X‐rays while reserving radiography for final confirmation [8].

No adverse events occurred within 24 h, supporting the safety of these nurse‐led techniques despite concerns about water/air boluses. Under a standardized protocol with vigilant monitoring, these methods do not compromise safety. The high PPV of palpation (95.7%) and perfect NPV of pH difference (100%) can guide clinical decision‐making during advancement. The combined palpation‐ultrasound approach achieved a PPV of 97.8%, suggesting that concordant positive results reliably predict correct placement, reducing unnecessary interim radiographs.

Based on these results, we recommend a tiered approach: (1) pH difference (≥ 1) for initial screening (perfect sensitivity); (2) palpation for confirmation (balanced accuracy); (3) ultrasound for challenging cases or when available. These methods serve as procedural aids only, not replacements for mandatory pre‐feeding radiographic confirmation per ASPEN guidelines [8].

### 6.1 Strengths and Limitations

Key strengths of this study include its prospective blinded design, which minimized bias; its standardized protocol, which ensured consistency; and the use of a single operator, which eliminated inter‐operator variability. To our knowledge, this is the first diagnostic accuracy study to directly compare palpation, ultrasound and pH difference against radiography for NET tip localization, providing novel evidence for nurse‐led techniques in resource‐limited settings. Inclusion of both placement outcomes enabled comprehensive evaluation.

### 6.2 Limitations

The single‐centre, single‐operator design limits generalizability. The small number of gastric placements (*n* = 13) led to imprecise specificity estimates (e.g., for pH difference) and reduced power for combined‐method comparisons. Procedural interventions (metoclopramide and water bolus) may limit applicability to other contexts. Although no adverse events occurred, safety in higher‐risk populations requires further evaluation.

### 6.3 Implications for Practice

This study supports the integration of three bedside methods—pH difference in aspirate, palpation for bubbling sounds and ultrasonographic gastric antrum assessment—into nurse‐led protocols for preliminary NET tip localization in resource‐limited ICUs. A stepwise approach is recommended: use pH difference (threshold ≥ 1) as an initial screening tool due to its 100% sensitivity; confirm placement with palpation, which offers balanced accuracy (PPV 95.7%); and reserve ultrasound for challenging cases. The combined use of palpation and ultrasound (PPV 97.8%) can reduce the need for interim radiographic verification. These methods are intended as procedural aids to guide placement and optimize resource use, not as substitutes for mandatory pre‐feeding radiographic confirmation. Implementation requires structured nurse training to ensure competency and adherence to safety protocols.

## 7 Conclusion

This study validates palpation, ultrasound and pH difference as accurate bedside methods for preliminary nasoenteric tube tip localization in resource‐limited ICUs. These tools may be integrated into nurse‐led protocols to reduce interim X‐rays while maintaining safety, pending multi‐centre validation.

## Author Contributions

Anshan Yu and Yanmei Xie equally contributed to the conception and design of the research. Mingfang Xiao contributed to the design of the research. Qingbang Zhong and Yueling He contributed to the acquisition and analysis of the data. Mingfang Xiao and Yanmei Xie contributed to the interpretation of the data. Qianhui Yu and Yanmei Xie drafted the article. All authors critically revised the article, agreed to be fully accountable for ensuring the integrity and accuracy of the work, and read and approved the final article.

## Funding

This work was supported by the Bureau of Science and Technology of Ganzhou Municipality (GZSTB; Ganzhou, Jiangxi Province, China; grant no.: GZ2021ZSF102).

## Ethics Statement

The study was approved by the Institutional Review Board of the First Affiliated Hospital of Gannan Medical University (reference number: LLSC‐2021101402) on 14 October 2021. Ethics approval was granted prior to study commencement.

## Consent

Written informed consent was obtained from all participants or their legally authorized representatives prior to inclusion in the study, in accordance with the ethical principles of the Declaration of Helsinki.

## Conflicts of Interest

The authors declare no conflicts of interest.

## Data Availability

The data that support the findings of this study are available on request from the corresponding author. The data are not publicly available due to privacy or ethical restrictions.
